# Correction: Rapid visual categorization is not guided by early salience-based selection

**DOI:** 10.1371/journal.pone.0226429

**Published:** 2019-12-11

**Authors:** John K. Tsotsos, Iuliia Kotseruba, Calden Wloka

Figs 4, 6 and 7 are incorrect. The authors have provided the corrected versions here.

**Fig 4:**
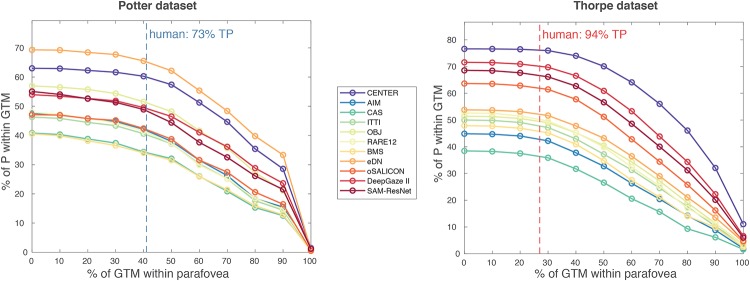
Sensitivity analysis of performance thresholds. The plots show how percentage of points of interest P within the ground truth masks and parafovea gradually decreases depending on how much of the GTM (by area) is inside the parafovea. On each plot the point (0,0) corresponds to measure B (i.e. P is within the GTM and parafovea regardless of the amount of GTM within the parafovea). Dashed vertical lines show what amount of overlap corresponds to human performance in Potter and Thorpe experiments.

**Fig 6:**
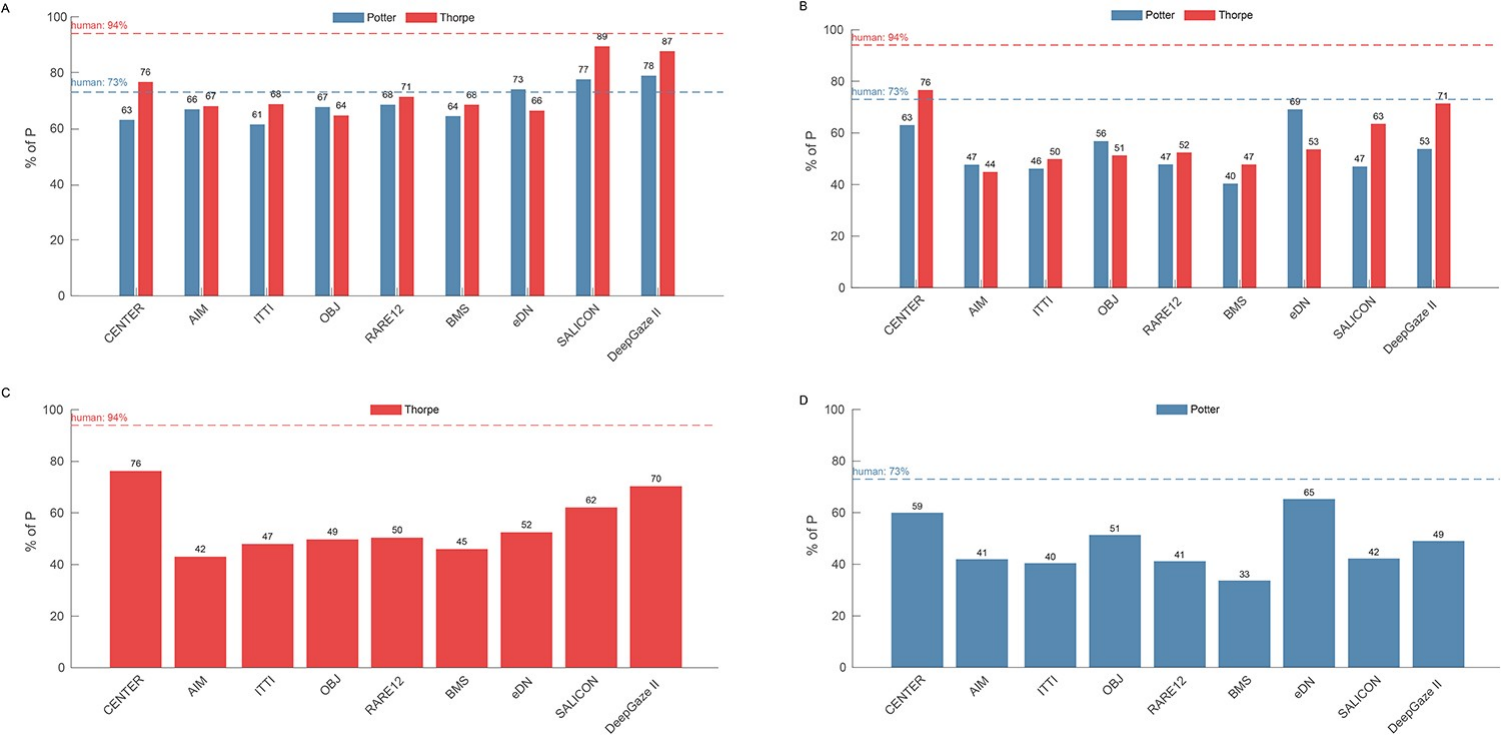
Plots of results using 4 performance measures. A: The percent of all first P that fall anywhere within the GTM for each tested algorithm and dataset. B: The percent of all first P that fall that fall within the GTM AND within the parafovea for each tested algorithm and dataset. C: The percent of all first P that fall within the GTM AND within the parafovea AND at least 27% of the GTM (by area) lies within the parafovea for each algorithm but for only the Thorpe images. D: The percent of all first P that fall within the GTM AND within the parafovea AND at least 41% of the GTM (by area) lies within the parafovea for each algorithm but only for the Potter images.

**Fig 7:**
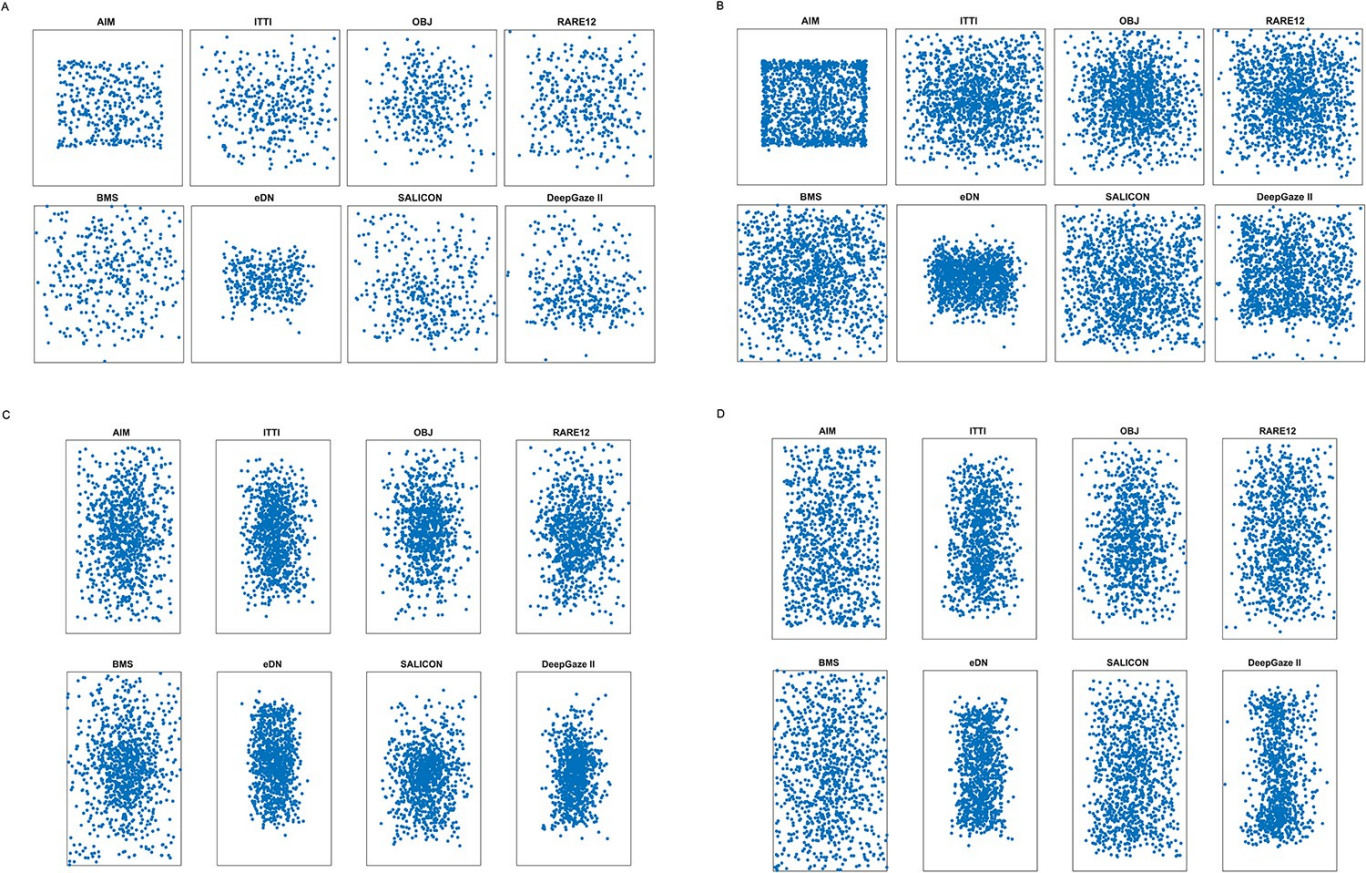
Scatterplots of the first attended locations P predicted by the saliency algorithms. A: P for target-present images in the Potter set. B: P for images with no target present from Potter set. C: P for target-present images in the Thorpe set. D: P for images with no target present from Thorpe set.
