# Supplementary figures and images for: Rapid visual categorization is not guided by early salience-based selection
Source: PLoS One. 2019 Oct 24;14(10):e0224306. doi: 10.1371/journal.pone.0224306 (PMC6812801; doi:10.1371/journal.pone.0224306)

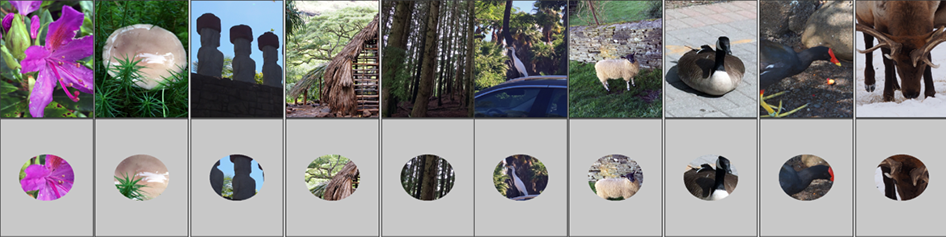

Supplement: S1 Fig — The top row shows full images used in the first experimental condition and the bottom row shows the same images cropped to the size of the parafovea, used in the second experimental condition. (TIF) [file pone.0224306.s001.tif]
