# Supplementary material for: Rapid visual categorization is not guided by early salience-based selection
Source: PLoS One. 2019 Oct 24;14(10):e0224306. doi: 10.1371/journal.pone.0224306 (PMC6812801; doi:10.1371/journal.pone.0224306)
Supplement: S1 Text — Links to the publicly available implementations of saliency algorithms used in this paper. (PDF) [file pone.0224306.s003.pdf]

S1 Text. Saliency models. The code for all saliency algorithms that we used is publicly available and was not modified for the experiments. Default parameters were used in each case. Below is a list of links.

AIM `https://github.com/TsotsosLab/AIM`.

ITTI implementation provided with the GBVS saliency package (`http://www.vision.caltech.edu/~harel/share/gbvs.php`).

OBJ v2.2 of the algorithm (`http://groups.inf.ed.ac.uk/calvin/objectness/`).

RARE2012 `http://www.tcts.fpms.ac.be/attention/?categorie17/rare2012`.

BMS we use the most recent version for eye-fixation prediction from [1] (`http://cs-people.bu.edu/jmzhang/BMS/BMS.html`).

eDN `https://github.com/coxlab/edn-cvpr2014`

oSALICON since the original algorithm is not published, we use the open source version of it that has comparable results as described in [2] (`https://github.com/CLT29/OpenSALICON`).

DeepGaze II `https://deepgaze.bethgelab.org/` (the code was not available at the time of the writing. We used the saliency maps provided by the authors to our data).

In all experiments we used default parameters.

OBJ outputs multiple object proposals as bounding boxes. These bounding boxes then can be combined to form a heatmap that approximately corresponds to a saliency map. The latter option was used in the experiments.

In order to evaluate inherent center bias of the algorithms we turned off the explicit center prior used in the following saliency models: ITTI, eDN and DeepGaze II. Other algorithms in our selection do not use the explicit center prior.

Even though we switched off the explicit center prior where applicable, there is still a possibility that some models, in particular the deep learning ones, may learn center bias from the training data. For example, the OSIE dataset [3] (used to train the oSALICON model), the oSALICON dataset [4] (used for the DeepGaze II model) and the MIT1003 dataset [5] (used for eDN) all have significant center bias.

References

1. Zhang J, Sclaroff S. Exploiting surroundedness for saliency detection: a boolean map approach. IEEE Transactions on Pattern Analysis & Machine Intelligence. 2016;(5):889–902.

2. Thomas C. OpenSALICON: An open source implementation of the salicon saliency model. arXiv preprint arXiv:160600110. 2016;.

3. Xu J, Jiang M, Wang S, Kankanhalli MS, Zhao Q. Predicting human gaze beyond pixels. Journal of vision. 2014;14(1):28–28.

4. Jiang M, Huang S, Duan J, Zhao Q. SALICON: Saliency in context. In: Proceedings of the IEEE Conference on Computer Vision and Pattern Recognition; 2015. p. 1072–1080.

5. Judd T, Durand F, Torralba A. A benchmark of computational models of saliency to predict human fixations; 2012.
