# Supplementary material for: Rapid visual categorization is not guided by early salience-based selection
Source: PLoS One. 2019 Oct 24;14(10):e0224306. doi: 10.1371/journal.pone.0224306 (PMC6812801; doi:10.1371/journal.pone.0224306)
Supplement: S2 Text — Justification of our focus on parafoveal region in the human experiment and analysis of saliency algorithms. (PDF) [file pone.0224306.s004.pdf]

S2 Text. Human retina characteristics. Our focus on the parafoveal region of a test image can be justified by considering the following. Sources of relevant information on photoreceptor distribution and other retinal characteristics in humans include [1], [2], and [3]. Without recounting all the details, it is well-known that density of retinal cones is at its maximum at the very center of the fovea and falls rapidly towards the periphery. At its center lies the foveola, 350μm wide (0.5 $^{\circ}$) that is totally rod-free and capillary free, thus seeming the optimal target for new visual information. The parafovea is the region immediately outside the fovea with a diameter of 2.5mm (5 $^{\circ}$). It is important to recall that acuity decreases with retinal eccentricity. Anstis [4] showed that to maintain visual acuity an object must increase by 2.76 arcmin in size for each degree of retinal eccentricity up to about 30 $^{\circ}$, and then somewhat more steeply up to 60 $^{\circ}$. It seems clear that if the target object that falls within the central 5 $^{\circ}$ of the retina, the likelihood of its correct categorization is much higher than if otherwise.

References

1. Østerberg G A. Topography of the layer of rods and cones in the human retina. Acta Ophthalmologica. 1935;6(Suppl 1):1–102.

2. Curcio CA, Sloan KR, Kalina RE, Hendrickson AE. Human photoreceptor topography. Journal of comparative neurology. 1990;292(4):497–523.

3. Curcio CA, Allen KA. Topography of ganglion cells in human retina. Journal of comparative Neurology. 1990;300(1):5–25.

4. Anstis SM. A chart demonstrating variations in acuity with retinal position. Vision research. 1974;14(7):589–592.
