# Supplementary material for: Rapid visual categorization is not guided by early salience-based selection
Source: PLoS One. 2019 Oct 24;14(10):e0224306. doi: 10.1371/journal.pone.0224306 (PMC6812801; doi:10.1371/journal.pone.0224306)
Supplement: S3 Text — Description of the human experiment and discussion of results. (PDF) [file pone.0224306.s005.pdf]

S3 Text. Experimental Details for the Parafoveal Stimuli Test. *Procedure.* There are two conditions in the study. In the first condition, we replicate the experiment conducted by Thorpe et al. [**?**]. In the experiment subjects view an image for the duration of 20ms and perform a go/no-go categorization where they have to decide whether an animal is present in the scene or not. Since no specific task definition was provided in the original description of the experiment, we have asked our participants to look for a live animals excluding humans. In addition, the subjects were instructed to ignore artistic renditions of animals, such as those corresponding to drawings, statues, etc.

In the second condition, the images were cropped so that only the area within the circle with radius of 2.5° (corresponding to the size of the parafovea) remained visible. Subjects were instructed to maintain fixation on the cross presented and to not move their eyes. Each trial begins with a fixation cross for 500ms, followed by an image for 20ms and a fixation cross, on a blank screen, for 500ms. The images are shown consecutively with a random interval of 1 to 2 seconds during which subjects have to press the space bar on the keyboard if they see an animal in the image.

Participants. A total of 17 subjects (6 women, 11 men), between the age of 25 and 34 years old, participated in the study. All participants were volunteers and were not compensated for their participation. Additionally, the participants were asked to sign a consent form approved by the York University Office of Research Ethics (Certificate number 2016-014 “Selective tuning approach to visual system attention executive”). Each subject completed 10 blocks of 100 images for each condition.

Materials. The stimuli were 2000 color photographs as used in the original experiment in Thorpe et al. The subjects were not familiar with the images and viewed each image only once. For each participant, the data was randomly split into two equally-sized sets for each experimental condition containing approximately the same number of images with and without animals. The images were resized to 256 by 384 pixels and were presented in the center of the monitor on a light gray background.

Apparatus. The experiments were programmed in Matlab R2016b using the Psychophysics Toolbox (Brainard 1997) version 3. The monitor, a ViewSonic Graphics Series GS815 19 in CRT, was set to 1024 x 768 resolution with 75 Hz refresh rate. All subjects were placed in a dark room and were seated 60 cm away from the monitor with their head movements restricted by a chin rest.

Results. The results of the first condition (full images) are similar to the ones reported by Thorpe et al. The average proportion of correct responses is 93% compared to the 94% in the original experiment. One of the subjects achieved the rate of 97% correct responses (98% in Thorpe et al.). In the second condition (cropped images) 85% of responses are correct with a maximum of 91% achieved by one of subjects. In the second condition we excluded trials where the target was located outside the parafovea region (overall $<1\%$ of the trials were removed as a result). Examples of full-size and cropped images used in the experiment are shown in S1 Fig.

We analyzed individual participants' percentages of correct responses as a function of the percentage of overlap between the parafovea mask ($r=2.5^\circ$) and a binary mask corresponding to the animal in the image. Only responses on target-present trials were considered for this analysis (mean responses of human subjects are provided as S1 File).

Examples for the full-size and cropped images are shown in top and bottom row of S1 Fig respectively. The results of the first experimental condition are shown in Fig 8A. Note that despite significant differences in individual performance, overall, most subjects have lower response accuracy when the target covers between 10 and 30% of

the parafovea. The accuracy levels out when the target occupies >40% of the parafovea.

There is much more variability in the subject responses in the second experimental condition (cropped images), shown in Fig 8B, because most of the context is not available (see S1 Fig bottom row). However, the same trend as in the first condition is still very noticeable. For instance, when the target occupies >20% of the parafovea, the average response accuracy is above 60%. Particularly, larger targets, covering >70% of the parafovea, were challenging because most of the animal was likely to be cropped out. Furthermore, in many cases, easily identifiable parts of the animals, such as head, wings, antlers, etc., are not necessarily present in the central region and for the images where the targets covered 100% of the parafovea, human/algorithm performance was a little worse.

We conducted a repeated-measures analysis of variance (ANOVA) on the human experimental data. The effect of target overlap with the parafovea on the accuracy of responses was significant in both conditions: $F(9, 144)=76.784 < 0.001$ and $F(9, 126)=100.305 < 0.001$ respectively.

Fig 8C shows the performance of the top 3 saliency algorithms (oSALICON, eDN and DeepGaze II). Note that this plot is only provided for qualitative comparison since it was not possible to subject the algorithms to the same experimental conditions as human participants. For instance, response times are not comparable due the fact that humans are required to make motor responses. Furthermore, we generously assume that the target is recognized by the algorithm if the maximum of the saliency map falls within the ground truth mask (Measure A). Therefore, results shown in Fig 8C should be interpreted as the upper bound on the accuracy of the saliency algorithms.

Several observations can be made based on the box plots shown in Fig 8. First, the variance of the responses for the saliency algorithms (Fig 8C) is much lower than that of the human subjects (Fig 8A,B). Since the saliency algorithms are trained on human data, they highlight similar features in the images, particularly faces of humans and animals. Second, the percentage of correct responses monotonically improves as the target size, with respect to the parafovea, increases. Essentially, the larger the target, the higher are the chances that saliency maxima will fall within the ground truth mask.

References

1. Thorpe S, Fize D, Marlot C. Speed of processing in the human visual system. Nature. 1996;381(6582):520–522.
